# Atrial fibrillation and anticoagulation are associated with hospitalisations in patients with end-stage kidney disease on haemodialysis: a prospective population-based cohort study

**DOI:** 10.1186/s12959-022-00434-7

**Published:** 2022-11-30

**Authors:** Daniel Steiner, Sabine Schmaldienst, Matthias Lorenz, Renate Klauser-Braun, Ingrid Pabinger, Cihan Ay, Marcus Säemann, Oliver Königsbrügge

**Affiliations:** 1 Clinical Division of Haematology and Haemostaseology, Department of Medicine I, Medical University of Vienna, Waehringer Guertel 18-20, A-1090 Vienna, Austria; 2 Department of Medicine I, Clinic Favoriten, Vienna, Austria; 3 Vienna Dialysis Centre, Vienna, Austria; 4 Department of Medicine III, Clinic Donaustadt, Vienna, Austria; 5 Department of Medicine VI, Clinic Ottakring, Vienna, Austria

**Keywords:** Haemodialysis, Atrial fibrillation, Anticoagulation, End-stage kidney disease, Hospitalisation

## Abstract

**Background:**

Patients with end-stage kidney disease on haemodialysis suffer from frequent complications requiring hospitalisation. Atrial fibrillation is a burdensome comorbidity amongst patients on haemodialysis. We aimed to assess frequency, reasons, and duration of hospitalisations in haemodialysis patients and their association with atrial fibrillation and anticoagulation.

**Methods:**

Prevalent patients with end-stage kidney disease on haemodialysis were recruited into a prospective cohort study and observed for a median observation time of 3.4 years. Hospitalisations were recorded from discharge letters, medical records, and patient interviews. The association of atrial fibrillation, anticoagulation, and time-in-therapeutic range of vitamin K antagonist treatment with hospitalisations was analysed using negative binomial regression.

**Results:**

Out of 625 patients, 238 (38.1%) had atrial fibrillation. Median number of hospitalisations per patient was 3.0 (1.0–5.0). Incidence rate of hospitalisation was 1.7 per patient-year in all and 1.9 in atrial fibrillation patients, median duration per hospitalisation was 7.9 (4.8–12.9) and 8.8 (5.7–13.3) days, respectively. Most frequent reasons for hospitalisation were vascular access complication/intervention (11.7%) and infection/fever (11.4%), while bleeding events comprised 6.0% of all hospitalisations. Atrial fibrillation patients had 27% higher risk of hospitalisation than patients without atrial fibrillation (incidence rate ratio [IRR] 1.27, 95% confidence interval [CI] 1.10–1.47). In atrial fibrillation patients, anticoagulation (enoxaparin or phenprocoumon, 41.6% of AF patients) was associated with increased risk of all-cause (IRR 1.38, 95%CI 1.14–1.69) and bleeding-related hospitalisation (IRR 1.96, 95%CI 1.06–3.63). There was no association between anticoagulation and stroke-related hospitalisation. In atrial fibrillation patients on phenprocoumon, increasing time-in-therapeutic range was associated with decreased risk of all-cause (IRR 0.35, 95%CI 0.14–0.87), but not bleeding-related hospitalisation (IRR 0.13, 95%CI 0.01–1.38).

**Conclusion:**

In haemodialysis patients, presence of atrial fibrillation and, among those with atrial fibrillation, anticoagulation were associated with higher risk of all-cause hospitalisation, including bleeding-related hospitalisation in the latter. Increasing time-in-therapeutic range in patients on vitamin K antagonist treatment was associated with decreased risk of all-cause, but not bleeding-related hospitalisation.

## Introduction

Patients with end-stage kidney disease (ESKD) on haemodialysis (HD) are vulnerable to exacerbated medical conditions that lead to a high risk of requiring repeated hospitalisation compared to patients without chronic kidney disease [1]. The high frequency of comorbid conditions in HD patients, the effects of HD on the immune system, and the overall increased age and frailty in the HD population predispose for complications that require inpatient treatment. These hospitalisations place a substantial burden on patients and health-care systems, since they are associated with adverse outcomes including mortality [2] and high costs, as shown for example by inflation-adjusted inpatient spending of $12.2B in the USA in 2019 for beneficiaries with ESRD [3].

Atrial fibrillation (AF) is a frequent comorbidity in HD patients, with a prevalence of up to 27% [4, 5], that significantly increases the risk of ischemic stroke, and anticoagulation for stroke prevention in AF is associated with increased risk of bleeding [6–9]. Beyond the risk of ischemic stroke and bleeding, patients with AF also exhibit a wide range of comorbidities [4]. The magnitude of the burden of hospitalisations in HD patients with AF compared to non-AF patients, the distribution of reasons for hospitalisation, and the impact of anticoagulation treatment on hospitalisation rates are unknown.

We aimed to prospectively assess the frequency, reasons, and duration of hospitalisations in patients undergoing maintenance HD in a large urban area in Austria. In a prespecified analysis, we aimed to compare the risk of hospitalisation in AF patients to non-AF patients. Furthermore, we aimed to investigate the effects of anticoagulation treatment and specifically the quality of vitamin-K-antagonist (VKA) treatment on the risk of hospitalisations.

## Materials and methods

### Study design and data collection

The Vienna InVestigation of Atrial fibrillation and thromboemboLism in hemoDIalysis patients (VIVALDI) is a prospective population-based cohort study, which was initiated to investigate the incidence of AF, thromboembolism, and bleeding in patients with ESKD on maintenance HD. It was approved by the local ethics committees (EC vote 1146/2014 and EK-14-099-0614) and was conducted according to international standards of Good Clinical Practice in accordance with applicable local regulations, the declaration of Helsinki, and institutional research policies and procedures.

Between April 2014 and July 2015, prevalent patients at seven HD centres in Vienna, Austria, were recruited in a cross-sectional fashion. With a population of 1.8 million, approximately 860 patients are receiving HD in Vienna; we approached 814 patients, of whom 626 provided written informed consent to participate in the study. In order to be eligible to participate, patients had to be 18 years or older, had to have kidney failure requiring maintenance HD, and had to have the ability to provide written informed consent. Exclusion criteria were pregnancy, including possible pregnancy, breast-feeding, incapability to consent, and hospitalisation at the time of enrolment. Each patient underwent structured interviews with trained study investigators. Medical records at the participating dialysis centres were reviewed and findings were verified with medical documentation and in consensus with the treating nephrologists.

Patients were prospectively followed for a maximum of 1350 days (45 months), and the occurrence of outcomes was assessed with one interim and one final personal interview and review of medical documentation at the dialysis centres. One male patient (0.16%) with AF was lost to follow-up.

### Definition of atrial fibrillation and anticoagulation exposure

A diagnosis of AF at inclusion was recorded for symptomatic or asymptomatic patients in any of the following: signs of atrial fibrillation on a recent 12-lead resting ECG within one month of recruitment; documented AF episode during previous ECGs conducted routinely or in case of arrhythmia at the dialysis centres; or recorded diagnosis of atrial fibrillation in medical records [4]. Patients were followed up on new diagnosis of atrial fibrillation throughout the study. All the patients who developed atrial fibrillation throughout study follow-up were included in the AF exposure group for the prespecified outcomes.

The approach to anticoagulation in AF patients and the choice of medication was based on the decision of the treating physician. Patients either received phenprocoumon, a vitamin K antagonist (VKA), or enoxaparin, a low molecular weight heparin, on non-HD days. The presence of anticoagulation treatment was assessed with chart and prescription review and personal verification with the patient. Patients underwent re-evaluation of anticoagulation at 1 year and at the end of the study. Due to treatment changes during follow-up, anticoagulation treatment was defined with an intention-to-treat approach. Intention-to-treat was considered in patients with AF and anticoagulation at inclusion with maintenance for at least 1 month or if ischemic stroke, transient ischemic attack, systemic embolism, myocardial infarction, cardiovascular death, or major bleeding occurred during the first month of observation while the patient was on anticoagulation [6]. In patients developing AF during follow-up, intention-to-treat was considered if the patient was started on anticoagulation at diagnosis and treatment was continued for 1 month or more [6].

Regarding systemic anticoagulation during HD sessions, 569 (91.0%) patients received low molecular weight heparin, 23 (3.6%) unfractionated heparin, 11 (1.8%) citrate dialysis, and 22 (3.5%) other agents (fondaparinux, argatroban).

### Outcomes

The prespecified primary objective of the study was to assess frequency, reasons, and duration of hospitalisations. The outcome, reasons for hospitalisation, was divided into 15 different categories, i.e., infection or fever; peripheral artery disease or amputation; vascular access complication or intervention; vascular access thrombosis; vascular access infection; dyspnoea or chronic obstructive pulmonary disease (COPD); fall, fracture or trauma; bleeding; cardiac event; arrhythmia; oncological reason; stroke or transient ischemic attack (TIA); venous thromboembolism (VTE); planned hospitalisation; and other reasons. Classification of hospitalisation into categories was based on interview data and medical documentation including discharge letters.

For the secondary objective, we investigated frequency, reasons, and duration of hospitalisations in the subgroup of AF patients; association between anticoagulation and hospitalisation for distinct reasons in AF patients; and association of increasing time-in-therapeutic range (TTR), as a measure for quality of anticoagulation control, with all-cause and bleeding-related hospitalisation in AF patients on phenprocoumon. For calculating TTR, we set the target international normalised ratio at 2.0 to 3.0 and used the linear interpolation/Rosendaal method [10].

### Statistical analysis

Baseline characteristics were reported as counts and proportions or quartiles, where appropriate. Differences in baseline characteristics between AF patients with and without anticoagulation therapy were assessed with the Mann-Whitney U test for non-parametric data and with the chi-squared test or the Fisher’s exact test, whenever appropriate, for binary outcomes. We recorded the incidence for the primary and secondary outcomes in the full cohort and calculated incidence rates. To assess the association of AF, anticoagulation use, and time-in-therapeutic international normalised ratio (INR) range with hospitalisations, we calculated incidence rate ratios and 95% confidence interval (CI) using negative binomial regression. All calculations were performed with IBM SPSS Statistics for Windows (Version 28.0, IBM Corp.) and Stata Statistical Software (Version 15.1, STATA Corp.).

## Results

Baseline patient characteristic of the full cohort (*n* = 625) and the subgroups of patients with and without atrial fibrillation (AF, *n* = 238; non-AF, *n* = 387), further stratified by anticoagulation treatment, are shown in Table 1. The median age in the full cohort was 66 years (25th -75th percentile, 54.5–75.0) and 394 (63.0%) were male. Coronary artery disease, diabetes, and hypertension were frequent comorbidities and nearly half of all patients were current or past smokers. In patients with AF, patients with phenprocoumon or enoxaparin therapy had significantly higher frequency of a history of stroke or TIA compared to patients without anticoagulation (39.3% and 36.8%, respectively, vs. 18.0%; *p* = 0.001 and 0.013, respectively). Furthermore, patients on phenprocoumon had a significantly higher frequency of a history of deep vein thrombosis, were significantly more likely to be current or past smokers and had a significantly lower rate of antiplatelet medication. Patients with enoxaparin were significantly more likely to have employment compared to AF patients without anticoagulation (18.4% vs. 4.3%, *p* = 0.008).

**Table 1:** Patient characteristics

	**All patients**	**Non-AF cohort**	**AF cohort**	**AF cohort by anticoagulation treatment**		
				**No anticoagulation**	**Phenprocoumon group**	**Enoxaparin group**
**Patients, n (%)**	625 (100)	387 (100)	238 (100)	139 (100)	61 (100)	38 (100)
**Male (%)**	394 (63.0)	235 (60.7)	159 (66.8)	89 (64.0)	42 (68.9)	28 (73.7)
**Age, median (25th -75th percentile)**	66.0 (54.5–75.0)	61.0 (48.0–71.0)	71.5 (64.0–79.0)	73.0 (62.0–80.0)	70.0 (63.5–76.0)	73.0 (67.8–77.3)
**BMI, median (25th -75th percentile)**	25.7 (22.4–29.5)	25.3 (22.1–29.4)	26.0 (22.9–29.6)	25.5 (22.4–29.7)	26.7 (24.3–30.2)	25.7 (22.9–28.0)
**Employment, n (%)**	56 (9.0)	42 (10.9)	14 (5.9)	6 (4.3)	1 (1.6)	7 (18.4)
**Time-in-therapeutic INR range, median % (25th -75th percentile)**	n.a.	n.a.	n.a.	n.a.	57.2 (39.5–72.3)	n.a.
**Aetiology of ESKD, n (%)**	-	-	-	-	-	-
**Diabetic NP**	160 (25.6)	97 (25.1)	63 (26.5)	35 (25.2)	20 (32.8)	8 (21.1)
**Vascular NP**	121 (19.4)	71 (18.3)	50 (21.0)	31 (22.3)	12 (19.7)	7 (18.4)
**Glomerulonephritis**	81 (13.0)	59 (15.2)	22 (9.2)	11 (7.9)	6 (9.8)	5 (13.2)
**Atrophic NP**	57 (9.1)	36 (9.3)	21 (8.8)	13 (9.4)	4 (6.6)	4 (10.5)
**Cystic nonhereditary NP**	36 (5.8)	19 (4.9)	17 (7.1)	11 (7.9)	3 (4.9)	3 (7.9)
**Hereditary NP**	31 (5.0)	21 (5.4)	10 (4.2)	7 (5.0)	1 (1.6)	2 (5.3)
**Nephrectomy**	20 (3.2)	9 (2.3)	11 (4.6)	4 (2.9)	6 (9.8)	1 (2.6)
**Toxic NP**	28 (4.5)	12 (3.1)	16 (6.7)	9 (6.5)	3 (4.9)	4 (10.5)
**Other causes**	91 (14.6)	63 (16.3)	28 (11.8)	18 (12.9)	6 (9.8)	4 (10.5)
**Dialysis history, n (%)**	-	-	-	-	-	-
**History of kidney transplantation**	90 (14.4)	57 (14.7)	33 (13.9)	16 (11.5)	8 (13.1)	9 (23.7)
**History of peritoneal dialysis**	46 (7.4)	29 (7.5)	17 (7.1)	9 (6.5)	5 (8.2)	3 (7.9)
**Dialysis parameters, median (25th -75th percentile)**	-	-	-	-	-	-
**Remaining diuresis, ml/day**	500 (0-1000)	500 (0-1000)	500 (0-1000)	500 (0-1000)	400 (0-1000)	200 (0-1000)
**Time on haemodialysis, years**	2.7 (1.0–5.0)	2.5 (1.0–5.0)	3.0 (1.0–6.0)	3.0 (1.0–6.0)	3.0 (1.7-7.0)	2.3 (1.0–5.0)
**Comorbidities, n (%)**	-	-	-	-	-	-
**History of stroke or TIA**	127 (20.3)	64 (16.5)	63 (26.5)	25 (18.0)	24 (39.3)	14 (36.8)
**History of myocardial infarction**	104 (16.6)	53 (13.7)	51 (21.4)	31 (22.3)	12 (19.7)	8 (21.1)
**Coronary artery disease**	232 (37.1)	121 (31.3)	111 (46.6)	62 (44.6)	29 (47.5)	20 (52.6)
**Artificial heart valve**	43 (6.9)	21 (5.4)	22 (9.2)	10 (7.2)	8 (13.1)	4 (10.5)
**History of VTE**	61 (9.8)	28 (7.2)	33 (13.9)	16 (11.5)	13 (21.3)	4 (10.5)
**Deep vein ** **thrombosis**	43 (6.9)	19 (4.9)	24 (10.1)	10 (7.2)	10 (16.4)	4 (10.5)
**Pulmonary ** **embolism**	32 (5.1)	16 (4.1)	16 (6.7)	7 (5.0)	8 (13.1)	1 (2.6)
**Peripheral artery disease**	197 (31.5)	111 (28.7)	86 (36.1)	48 (34.5)	20 (32.8)	18 (47.4)
**Diabetes**	237 (37.9)	135 (34.9)	102 (42.9)	63 (45.3)	28 (45.9)	11 (28.9)
**Hypertension**	574 (91.8)	355 (91.7)	219 (92.0)	128 (92.1)	57 (93.4)	34 (89.5)
**Congestive heart failure**	183 (29.3)	91 (23.5)	92 (38.7)	49 (35.3)	27 (44.3)	16 (42.1)
**Cancer history or active**	152 (24.3)	76 (19.6)	76 (31.9)	44 (31.7)	19 (31.1)	13 (34.2)
**Current and past smokers**	305 (48.8)	191 (49.4)	114 (47.9)	64 (46.0)	37 (60.7)	13 (34.2)
**History of major bleeding**	67 (10.7)	38 (9.8)	29 (12.2)	20 (14.4)	5 (8.2)	4 (10.5)
**History of intracranial bleeding**	18 (2.9)	8 (2.1)	10 (4.2)	7 (5.0)	2 (3.3)	1 (2.6)
**CHA2DS2-VASc score, median (25th -75th percentile)**	4 (2–5)	4 (3–5)	4 (3–5)	4 (3–5)	4 (3–5)	4 (3–5)
**HAS-BLED score, median (25th -75th percentile)**	3 (2–4)	4 (3–4)	4 (3–4)	4 (3–4)	3 (2–4)	4 (3–5)
**Antiplatelet medication**	345 (55.2)	208 (53.7)	137 (57.6)	98 (70.5)	18 (29.5)	21 (55.3)

*AF* Atrial fibrillation, *BMI* Body mass index, *INR* International normalised ratio, *ESKD* End-stage kidney disease, *NP* Nephropathy, *TIA* Transient ischemic attack, *VTE* Venous thromboembolism

### Reasons, frequency and duration of hospitalisations

During a median (25th -75th percentile) observation time of 3.4 (2.9–3.5) years, the incidence rate of hospitalisation was 1.7 per patient year across all patients. Incidence rate, duration of hospital stays and frequency of hospitalisation by reason are shown in Table 2. The median number of hospitalisations per patient was 3.0 (1.0–5.0) and the median duration per hospitalisation was 7.9 days (4.8–12.9). Most frequent reasons for hospitalisation were vascular access complication or intervention, infection or fever, and peripheral artery disease or amputation. Bleeding events, arrhythmia and stroke comprised 6.0%, 3.3% and 1.3% of all hospitalisations, respectively. While vascular access complication or intervention was the most frequent reason for hospitalisation, patients spent nearly thrice as many days for infection or fever in a hospital (1,338 vs. 3,735 days). Stroke or TIA had the longest median hospital stay durations, with a median of 12.0 days (25th -75th percentile, 6.0-24.5).

**Table 2:** Reasons, incidence rates, duration, and number of hospitalisations in the full cohort

**Reason for hospitalisation (full cohort, )** ***n*** ** = 625)**	**Incidence of hospitalisation per 100 patient years for this reason**	**Median (25th to 75th percentile) days per hospitalisation for this reason**	**Sum of hospitalisations for this reason**	**Percentage of all hospitalisations**	**Sum of days hospitalized for this reason**	**Percentage of days hospitalized**
**All-cause hospitalisation**	165.7	7.9 (4.8–12.9)	2333	100	24,921	100
**VA complication/intervention**	19.3	3.5 (2.1–5.5)	272	11.7	1338	5.4
**Infection/fever**	19.0	9.5 (6.0-15.3)	267	11.4	3735	15.0
**PAD/amputation**	13.0	10.5 (4.0-23.6)	183	7.8	3326	13.4
**Dyspnoea/COPD**	10.4	7.0 (4.0–12.0)	146	6.3	1293	5.2
**Fall/fracture/trauma**	10.1	9.2 (4.0–21.0)	142	6.1	2110	8.5
**Planned hospitalisation**	10.0	3.0 (2.0–4.0)	141	6.0	505	2.0
**Bleeding**	9.9	6.5 (4.0–17.0)	139	6.0	1647	6.6
**Cardiac event**	8.9	7.0 (3.5–14.0)	125	5.4	1729	7.0
**VA thrombosis**	8.3	5.0 (3.0–9.0)	117	5.0	863	3.5
**Arrhythmia**	5.5	8.0 (3.0–15.0)	77	3.3	865	3.5
**Oncological reason**	4.3	6.5 (3.1–11.8)	60	2.6	491	2.0
**VA infection**	3.5	10.5 (7.0-15.5)	49	2.1	783	3.1
**Stroke/TIA**	2.1	12.0 (6.0-24.5)	30	1.3	474	1.9
**VTE**	0.4	10.0 (5.3–18.5)	6	0.3	63	0.3
**Other reasons**	41.1	7.0 (4.0–12.0)	579	24.8	5699	22.9

*VA* Vascular access, *PAD* Peripheral artery disease, *COPD* Chronic obstructive pulmonary disease, *TIA* Transient ischemic attack, *VTE* Venous thromboembolism

In AF patients, the incidence rate of hospitalisation was 1.9 per patient year, the median (25th -75th percentile) number of hospitalisations was 3.5 (2.0–6.0) and the median duration per hospitalisation was 8.8 (5.7–13.3) days. Patients with AF had 27% higher risk of hospitalisation than non-AF patients (incidence rate ratio [IRR] 1.27, 95% confidence interval [CI] 1.10–1.47, *p* = 0.001). In both patients with and without AF, most frequent reasons for hospitalisation were infection or fever, vascular access complication or intervention, and peripheral artery disease or amputation, albeit in a differing order. Compared to patients without AF, patients with AF had higher incidence rates for bleeding-related, arrhythmia-related and stroke-related hospitalisation. Incidence rate, duration of hospital stays and frequency of hospitalisation by reason in AF patients compared to non-AF patients are shown in Table 3.

**Table 3:** Reasons, incidence rates, duration, and number of hospitalisations stratified by presence of atrial fibrillation

**Reason for hospitalisation stratified by presence of AF (AF *n*** = 238, non-AF *n*** = 387)**	**Incidence of hospitalisation per 100 patient years for this reason**		**Median (25th to 75th percentile) days per hospitalisation for this reason**		**Sum of hospitalisations for this reason**		**Percentage of all hospitalisations**		**Sum of days hospitalized for this reason**		**Percentage of days hospitalized**	
	**AF**	**non-AF**	**AF**	**non-AF**	**AF**	**non-AF**	**AF**	**non-AF**	**AF**	**non-AF**	**AF**	**non-AF**
**All-cause hospitalisation**	186.5	153.0	8.8 (5.7–13.3)	7.2 (4.6–12.6)	998	1335	100	100	10,668	14,253	100	100
**VA complication/** **intervention**	19.8	19.0	3.5 (2.0–5.0)	3.8 (2.7-6.0)	106	166	10.6	12.4	472	866	4.4	6.1
**Infection/fever**	22.8	16.6	11.0 (6.3–15.7)	9.0 (6.0-14.5)	122	145	12.2	10.9	1685	2050	15.8	14.4
**PAD/amputation**	17.9	10.0	11.0 (3.6–29.3)	10.0 (5.0-22.9)	96	87	9.6	6.5	1811	1515	17.0	10.7
**Dyspnoea/COPD**	13.5	8.5	6.0 (3.8–11.3)	8.0 (4.3–12.2)	72	74	7.2	5.5	625	668	5.9	4.7
**Fall/fracture/** **trauma**	12.0	8.9	8.0 (4.5–21.5)	9.5 (4.0–21.0)	64	78	6.4	5.8	925	1185	8.7	8.3
**Planned hospitalisation**	11.2	9.3	3.0 (2.0–4.0)	3.0 (2.0–4.0)	60	81	6.0	6.1	238	267	2.2	1.9
**Bleeding**	10.8	9.3	7.0 (4.0–15.0)	6.0 (4.0–19.0)	58	81	5.8	6.1	626	1021	5.9	7.2
**Cardiac event**	8.6	9.1	9.0 (3.5–19.0)	6.0 (3.2–12.8)	46	79	4.6	5.9	635	1094	6.0	7.4
**VA thrombosis**	7.1	9.1	7.0 (4.0-9.5)	5.0 (3.0-8.1)	38	79	3.8	5.9	313	550	2.9	3.9
**Arrhythmia**	11.2	1.9	6.3 (3.0-11.6)	13.0 (4.5–28.0)	60	17	6.0	1.3	525	340	4.9	2.4
**Oncological reason**	4.7	4.0	5.5 (2.0-13.3)	6.5 (3.8–11.8)	25	35	2.5	2.6	229	262	2.1	1.8
**VA infection**	3.6	3.4	10.0 (5.0-15.5)	11.0 (8.0-15.5)	19	30	1.9	2.2	320	463	3.0	3.3
**Stroke/TIA**	2.8	1.7	9.0 (6.0–25.0)	16.0 (6.0–25.0)	15	15	1.5	1.1	230	244	2.2	1.7
**VTE**	0.6	0.3	-	-	3	3	0.3	0.2	35	28	0.3	0.2
**Other reasons**	40.0	41.8	7.5 (4.0–12.0)	7.0 (4.0-12.1)	214	365	21.4	27.3	1999	3700	18.7	26.0

*AF* Atrial fibrillation, *VA* Vascular access, *PAD* Peripheral artery disease, *COPD* Chronic obstructive pulmonary disease, *TIA* Transient ischemic attack, *VTE* Venous thromboembolism

### Hospitalisations in AF patients in regard to anticoagulation therapy

Of all patients with AF, 61 and 38 patients received phenprocoumon and enoxaparin, respectively (41.6% of all AF patients). Anticoagulation was associated with an increased risk of all-cause (IRR 1.38, 95%CI 1.14–1.69, *p* = 0.001) and bleeding-related hospitalisation (IRR 1.96, 95%CI 1.06–3.63, *p* = 0.031, Table 4). There was no association between anticoagulation and stroke/TIA-related hospitalisation. In AF patients on phenprocoumon, increasing TTR was associated with significantly decreased risk of all-cause (IRR 0.35, 95%CI 0.14–0.87, *p* = 0.025), but not bleeding-related hospitalisation (IRR 0.13, 95%CI 0.01–1.38, *p* = 0.09).

**Table 4:** Incidence rate ratios for hospitalisations and hospitalised days in AF-patients with vs. without anticoagulation

Reasons for hospitalisation	IRR for number of hospitalisations	*p*-value	IRR for number of hospitalised days	*p*-value
**All-cause hospitalisation**	1.38 (1.14–1.69)	**0.001**	1.57 (1.16–2.12)	**0.003**
**VA complication/intervention**	1.04 (0.62–1.75)	0.875	1.41 (0.67–2.97)	0.362
**Infection/fever**	1.50 (0.93–2.41)	0.093	1.64 (0.70–3.85)	0.258
**PAD/amputation**	0.98 (0.49–1.99)	0.965	1.79 (0.54–5.90)	0.340
**Dyspnoea/COPD**	1.74 (0.85–3.57)	0.128	1.02 (0.32–3.22)	0.973
**Fall/fracture/trauma**	1.85 (0.93–3.70)	0.081	2.38 (0.72–7.88)	0.155
**Planned hospitalisation**	1.14 (0.64–2.04)	0.658	2.08 (0.84–5.17)	0.113
**Bleeding**	1.96 (1.06–3.63)	**0.031**	1.20 (0.38–3.74)	0.753
**Cardiac event**	1.80 (0.80–4.04)	0.156	3.33 (0.80–13.9)	0.097
**VA thrombosis**	0.77 (0.29–2.02)	0.594	1.27 (0.28–5.80)	0.758
**Arrhythmia**	1.12 (0.60–2.13)	0.700	0.73 (0.26–2.08)	0.558
**Oncological reasons**	2.97 (1.03–8.56)	**0.044**	5.55 (0.91–33.9)	0.064
**VA infection**	1.52 (0.55–4.17)	0.418	2.99 (0.43–20.8)	0.268
**Stroke/TIA**	-	-	0.23 (0.03–1.81)	0.163
**VTE**	0.78 (0.01–109.1)	0.922	0.07 (0.00–40.3)	0.406
**Other reasons**	1.47 (1.05–2.07)	**0.025**	1.48 (0.82–2.65)	0.189

Bold values signify statistical significance

*IRR* Incidence rate ratio, *VA* Vascular access, *PAD* Peripheral artery disease, *COPD* Chronic obstructive pulmonary disease, *TIA* Transient ischemic attack, *VTE* Venous thromboembolism

## Discussion

In this population-based, prospective cohort study which assessed nearly three quarters of all prevalent patients on HD in Vienna, we showed an incidence rate of 1.7 hospitalisations per patient year with a median duration of 7.9 days. Most frequent reasons for hospitalisation included vascular access complication or intervention, infection or fever, and peripheral artery disease or amputation. Although most frequent reasons for hospitalisation were the same in the subgroup of AF patients, these patients had a significantly higher risk of all-cause hospitalisation. Anticoagulation with phenprocoumon and enoxaparin in AF patients was associated with increased risk of all-cause and bleeding-related hospitalisation, but there was no association with stroke/TIA-related hospitalisation. Increasing TTR in AF patients on phenprocoumon decreased the risk of all-cause hospitalisation. However, it was not associated with a decreased risk of bleeding-related hospitalisation.

Our results regarding incidence rate of hospitalisations are in line with those reported in the most recent annual report from the United States Renal Data System (USRDS) [3]. Adjusted rates of patients on haemodialysis in 2019 were 1.64 per patient year for hospitalisations and 1.99 for hospitalisations and observation hospital stays, the latter being considered outpatient services. Slightly lower rates have been shown in a retrospective cohort study in Canada, with 1.40, 1.35, and 1.18 hospitalisations per patient year at 7 days, 30 days, and 6 months after starting dialysis, respectively, in patients age 65 years or older [11]. The authors reported similar results for all other age groups except for paediatric patients, who had higher rates. Paediatric patients were excluded from our study. The lower rates of hospitalisations in the other age groups might be partly attributed to the fact that patients in our study had been on HD prior to inclusion and showed considerably more comorbidities. In the Dialysis Outcomes and Practice Patterns Study (DOPPS), a prospective, observational, multinational study, hospitalisation rate per patient year ranged from 0.78 in Italy to 1.58 in France [12]. The authors suggest that these differences might be attributed to the varying organization of in-patient care within countries. Worldwide differences which affect outcomes in patients with ESKD are extensively described in a Series paper by Robinson et al. [13]. While the incidence rate of France in the DOPPS study is comparable to our results, the median length of hospital stay is markedly lower with 4 days. However, median stays in Italy, Spain, and Germany were 7, 7, and 10 days, respectively, therefore similar to our results [12].

With regard to reasons for hospitalisation, the USRDS shows that hospitalisation for cardiovascular reasons is the most frequent cause, with 0.44 admissions per patient year in 2019 [3]. This result is difficult to compare with our study, since we provide specific results for peripheral artery disease or amputation, cardiac events, stroke or TIA, and VTE. Furthermore, there might be differences in data collection and classification, since we based our analysis on interview data and medical documentation including discharge letters and not on ICD codes. While we found a rate of 0.19 hospitalisations for vascular access complications or interventions per patient year, the USRDS reports 0.14 for vascular access reasons [3]. Similar to our study, the DOPPS study showed that between 11% and 17% of all hospitalisations are attributable to infections and septicaemia, with rates varying for different countries [12]. In contrast, the authors reported rates between 0.09 and 0.17 per patient year for gastrointestinal or liver-related hospitalisations, while we did not report results for this subcategory [12]. This difference might be partly attributed to differences in assessment method, which the authors of the DOPPS study did not describe in detail, and to misclassification of hospitalisations for this reason in the category for other hospitalisations in our study. In a recent analysis of the Chronic Renal Insufficiency Cohort (CRIC) study, unadjusted all-cause hospitalisations rate was 35.0 per 100 patient years in nearly 4000 patients with chronic kidney disease [14] The authors further report frequencies of primary cause of each hospitalisation, with diseases of the circulatory system being the most common by far, followed by disease of the genitourinary system and the digestive system [14]. However, comparisons to our study are difficult to draw, since reasons for hospitalisations were assessed with ICD-9 or ICD-10 admission and classified into categories with the Clinical Classification System software by the Agency for Healthcare Research and Quality [15] and, more importantly, the CRIC study excluded patients with prior dialysis [16, 17].

To our knowledge, there is no study evaluating hospitalisations in the subgroup of patients on haemodialysis with AF and associating anticoagulation use with cause-specific hospitalisations for these patients. Anticoagulation with VKA in HD patients is a controversial topic, owing to its potential impact on vascular calcification [18] and calciphylaxis [19], the high prevalence of functional vitamin K deficiency in HD patients [20] and pharmacokinetic issues which complicate targeting the therapeutic range [21]. Furthermore, systematic reviews and meta analyses evaluating observational studies of anticoagulation with warfarin have shown high bleeding rates [22] and non-favourable outcomes in AF patients specifically, with no effect on risks of ischemic stroke and mortality, but increased risk of major bleeding [23, 24]. In our study, the VKA phenprocoumon was used in about a quarter of all patients with AF. Alternatively, patients were able to receive enoxaparin, a low molecular weight heparin, on non-HD days, which is a pragmatic approach for patients with contraindications to or refusal of VKA therapy. Anticoagulation in AF patients was associated with significantly increased risk of all-cause and bleeding-related hospitalisation, which are patient-relevant outcomes that place a substantial additional burden on affected patients. Since the other cause-specific hospitalisations were not associated with anticoagulation, except for other unclassified reasons, this result seems to be primarily driven by bleeding events. This further adds to the controversy whether AF patients on HD derive a benefit from being anticoagulated. The role of direct oral anticoagulants (DOACs) in these patients was evaluated by observational studies [25–27] and one randomized, controlled trial [28], but since the use of these agents is off-label and not reimbursable in Austria, we cannot provide data on this topic.

As mentioned before, targeting the therapeutic range in AF patients on HD who are taking VKA is clinically challenging. Studies in AF patients in general taking warfarin have shown an association between higher TTR and better outcomes, and vice versa [29, 30]. This included an association between out of target INR and rate of hospitalisations in a record linkage study of over 2000 patients [30]. While it might not be universally possible to extrapolate these results to AF patients on HD, it seems reasonable that this association is similar to some extent in these patients. In our cohort, median TTR was about 57% and increasing TTR was associated with significantly decreased risk of all-cause hospitalisation. This association was not shown for bleeding-related hospitalisation which might be attributed to the limited sample size in this subgroup and the fact that out of target INR encompassed both sub- and supratherapeutic values. However, these results underline the potential value of TTR in AF patients on HD.

Hospitalisations in HD patients potentially pose a considerable burden on individual patients and the society as a whole, thus representing an important outcome. Therefore, hospitalisations should be considered as an outcome measures in clinical studies.

Our study has various limitations and strengths. Firstly, the observational nature of this study with the relatively small sample size, especially in the subgroups, is prone to bias. However, we were able to include a considerable proportion of all HD patients in a large metropolitan area with negligible loss to follow-up. Secondly, our approach to defining the cohorts possibly bears the risk of misclassification based on exposure. However, it is arguable that patients without atrial fibrillation at inclusion who developed it throughout the study had the predisposition for atrial fibrillation in the first place, thus placing them in the exposure cohort. Furthermore, different types of atrial fibrillation, i.e. paroxysmal or persistent, may present in a different manner. A patient with paroxysmal atrial fibrillation may remain undiagnosed until a symptomatic episode with ECG confirmation. Therefore, we believe that our approach is valid. We based the assessment of hospitalisations on personal interviews and medical documentations, not on ICD codes or billing claims. This approach allows for specification of reasons, e.g., different vascular-access related hospitalisations, but might lead to some extent of misclassification. While a large proportion of hospitalisations could not be classified into one of the prespecified reasons, this could partly be explained by the variability and diversity of reasons for which patients on HD are hospitalized. Furthermore, we only assessed primary reasons for hospitalisations and did not take into account the clinical course and complications, leading to a potential overlap in reasons for hospitalisations. In patients presenting with multiple medical issues, we aimed at assessing the most severe problem which influenced the decision for hospitalisation the most. However, there remains a residual risk of misclassification. The cohort was recruited and observed before the emergence of COVID-19, which has gravely struck the HD population [31]. Our results are therefore unaffected by COVID-19. In addition, our cohort did not include patients taking DOACs due to the specific situation of reimbursement in Austria, therefore it is not possible to make assumptions for these agents. Lastly, the approach to anticoagulation in AF patients and the choice of medication was not randomized and based on the decision of the treating physician, possibly influenced by preferences of patients, life expectancy, and anticipated adherence.

## Conclusion

In our prospective study of HD patients, we showed that hospitalisations place a substantial burden on this population, with vascular access complication or intervention, infection or fever, and peripheral artery disease or amputation being the most common reasons. This burden was amplified in patients with AF, who were more likely to be hospitalized and had a longer median duration per hospitalisation. Anticoagulation in these patients was associated with worse outcomes regarding all-cause and bleeding-related hospitalisations, without protection from stroke/TIA-related hospitalisation. In patients on VKA, TTR might be associated with hospitalisations, although this association warrants further evaluation.

## Data Availability

Data cannot be shared because Austrian law forbids the sharing of primary patient data.

## Abbreviations

AF: Atrial fibrillation
CI: Confidence interval
COPD: Chronic obstructive pulmonary disease
CRIC: Chronic Renal Insufficiency Cohort
DOPPS: Dialysis Outcomes and Practice Patterns Study
ESKD: End-stage kidney disease
HD: Haemodialysis
ICD: International Classification of Diseases
INR: International normalised ratio
IRR: Incidence rate ratio
PAD: Peripheral artery disease
TIA: Transient ischemic attack
TTR: Time-in-therapeutic range
USRDS: United States Renal Data System
VA: Vascular access
VKA: Vitamin K antagonist
VTE: Venous thromboembolism
